# Inferring Epitopes of a Polymorphic Antigen Amidst Broadly Cross-Reactive Antibodies Using Protein Microarrays: A Study of OspC Proteins of *Borrelia burgdorferi*

**DOI:** 10.1371/journal.pone.0067445

**Published:** 2013-06-24

**Authors:** Elisabeth Baum, Arlo Z. Randall, Michael Zeller, Alan G. Barbour

**Affiliations:** 1 Division of Infectious Diseases, Department of Medicine and Department of Microbiology & Molecular Genetics, University of California - Irvine, Irvine, California, United States of America; 2 Institute for Genomics and Bioinformatics, School of Information and Computer Sciences, University of California - Irvine, Irvine, California, United States of America; Cornell University, United States of America

## Abstract

Epitope mapping studies aim to identify the binding sites of antibody-antigen interactions to enhance the development of vaccines, diagnostics and immunotherapeutic compounds. However, mapping is a laborious process employing time- and resource-consuming ‘wet bench’ techniques or epitope prediction software that are still in their infancy. For polymorphic antigens, another challenge is characterizing cross-reactivity between epitopes, teasing out distinctions between broadly cross-reactive responses, limited cross-reactions among variants and the truly type-specific responses. A refined understanding of cross-reactive antibody binding could guide the selection of the most informative subsets of variants for diagnostics and multivalent subunit vaccines. We explored the antibody binding reactivity of sera from human patients and *Peromyscus leucopus* rodents infected with *Borrelia burgdorferi* to the polymorphic outer surface protein C (OspC), an attractive candidate antigen for vaccine and improved diagnostics for Lyme disease. We constructed a protein microarray displaying 23 natural variants of OspC and quantified the degree of cross-reactive antibody binding between all pairs of variants, using Pearson correlation calculated on the reactivity values using three independent transforms of the raw data: (1) logarithmic, (2) rank, and (3) binary indicators. We observed that the global amino acid sequence identity between OspC pairs was a poor predictor of cross-reactive antibody binding. Then we asked if specific regions of the protein would better explain the observed cross-reactive binding and performed *in silico* screening of the linear sequence and 3-dimensional structure of OspC. This analysis pointed to residues 179 through 188 the fifth C-terminal helix of the structure as a major determinant of type-specific cross-reactive antibody binding. We developed bioinformatics methods to systematically analyze the relationship between local sequence/structure variation and cross-reactive antibody binding patterns among variants of a polymorphic antigen, and this method can be applied to other polymorphic antigens for which immune response data is available for multiple variants.

## Introduction

Exploitation of the specificity of antibodies’ recognition of antigenic targets is the core of immunodiagnostic, immunotherapeutic and vaccine technologies. B-cell epitopes, which are recognized by antibodies or B-cells, can be divided into linear or conformational. For linear epitopes of polypeptides, the binding site is typically 10–15 contiguous residues on the antigen’s molecule [1], whereas conformational epitopes may be formed by residues that are brought together in 3-dimensional surface of the antigen. Epitopes may be unique or conserved amongst several antigenic targets. Epitope mapping studies aim to identify these binding sites so that antibody-antigen interactions of interest can be isolated to enhance the development of vaccines, diagnostics and immunotherapeutic compounds. However, the mapping of epitopes for antibodies is a time- and resource-consuming technique, employing synthesis of overlapping peptides, controlled proteolysis, or genetic manipulations of the encoding sequence that yield amino acid substitutions, deletions, or polypeptide truncations. Another, potentially more rapid and cost-effective approach is the use of epitope prediction programs that utilize information derived from primary amino acid sequence or its known or predicted secondary and tertiary structures [2]–[4].

A different challenge is cross-reactivity between epitopes, that is, those shared between two or more antigens, which otherwise can be distinguished by their type-specific epitopes. Meeting this challenge means teasing out the distinctions between broadly cross-reactive responses, limited cross-reactions among clusters of variants of the same protein, and the truly type-specific responses. More refined understanding of cross-reactive antibody binding between polymorphic antigens could guide the process of selecting the most informative subsets of variants for diagnostics and multivalent subunit vaccines. But is it possible to parse out the limited cross-reactivity from the broad cross-reactive responses?

One suitable model system to explore these issues is the binding of antibodies to the highly polymorphic protein OspC of the Lyme disease (LD) agent *Borrelia burgdorferi*. OspC is a surface-exposed lipoprotein that elicits an immunodominant antibody response early in infection [5]–[8]. There are at least 25 types of OspC proteins represented in the U.S. as a whole, though the number of *ospC* genotypes prevalent in any given geographic area range between 10 and 15 [9]. After conserved N-terminal signal peptide is cleaved, amino acid sequence identities for all pairs of known OspC types are between 63% to 90% [9], [10]. In experimental animal infections immunization with purified OspC provides protection against challenge [11]–[16] but usually only for the strain expressing the same OspC type [8], [12], [14]–[18].

Despite this evidence of OspC–type specific immunity and for type-specific epitope antibodies, a single OspC type in immunodiagnostic assay preparations has provided for reasonably good sensitivity [19]–[21]. This performance level is attributable to cross-reactivity in OspC proteins, especially when they are presented as isolated polypeptides on matrices such as blot membranes or microtiter plates [22], [23]. However, the sensitivity of OspC-based assays could plausibly be improved by the inclusion of multiple OspC proteins, ones that more fully represent the diversity of types that at-risk humans are likely to encounter [21], [24]. An equally desirable feature for an OspC-based immunodiagnostic assay would instead take advantage of strain-specific epitopes to discern the infecting strain of *B. burgdorferi*. This inference would be potentially useful for clinical management because *B. burgdorferi* strains, which are definable by their *ospC* genotypes [25], differ in their propensities to disseminate in the body, thus contributing to different disease manifestations in patients [26]–[29] and experimental models [30], [31].

Our approach to this challenge began with development of a protein microarray displaying purified recombinant proteins of several naturally-occurring variants of OspC in North America. Microarrays have been used to probe immune responses to proteomes of several human pathogens [32]–[35] including *B. burgdorferi* [36]. We obtained a panel of sera from LD patients and exposed it to the OspC variants on the array, and the resulting experimental data was used to quantify the degree of cross-reactive antibody binding between all pairs of variants. The goal was to relate these data to the amino acid sequence variation between OspC pairs to identify the region of the protein molecule most likely responsible for the cross-reactivity observed. For this aim, we developed a systematic computational analysis of the relationship between the cross-reactivity data and variation in subsets of either linear sequences or predicted 3-dimensional structures. These data and analyses provide a comprehensive study of cross-reactivity of antibody binding to an immunodominant protein antigen.

Notations and abbreviations used throughout this article are detailed in Text S1.

## Results

### Characterization of Sera from Patients with LD and from Controls

The 55 patient sera comprised 12 samples from early LD, 25 samples from patients with disseminated and late disease stages, and 18 samples from LD patients with persistent oligoarticular arthritis. All were seropositive by standard criteria for the diagnosis of LD by whole-cell ELISA and then confirmatory immunoblot. Sera from patients were significantly more reactive than sera from controls against each of several antigens. The mean (95% confidence intervals) for array binding in pixels per spot for patient sera and control sera were 11,748 (10,000 to 13,803) and 2,818 (1,995 to 3,981) for the B31 strain whole cell lysate; 1,905 (1,122 to 3,235) and 114 (91 to 147) for Decorin Binding Protein B (DbpB); 5,011 (3,467 to 7,244) and 416 (251 to 691) for the flagellin FlaB; and 2,818 (2,041 to 3,890) and 977 (776 to 1,230) for the VlsE outer membrane protein, respectively. Table S1 lists the results of the binding of antibodies of patients and controls to other *B. burgdorferi* antigens.

The protein microarray developed for this study displayed 23 different OspC variants including types K, A, B, N, and U, which were the most prevalent in nymphal ticks in the northeastern U.S. in a recent survey [25]. OspC types also included I, H, C and M, which are associated with more invasive infections [26]–[28]. Overall, sera from LD patients had significantly higher antibody binding to OspC proteins than naïve controls. The mean (95% confidence interval) binding intensity to all OspC spots was 1,406 (1,135 to 1,677) and 76 (66 to 87) for patient and control sera, respectively. Figure S1 summarizes the degree of antibody binding to each OspC protein by sera of LD patients or the control group. The raw quantitative output of pixel intensity of antibody binding to OspC proteins on the microarray by the sera sets used in this study is provided in Table S2, along with their respective log_10_, rank and binary transforms.

### Cross-reactivity among OspC Variants

**Figure 1:**
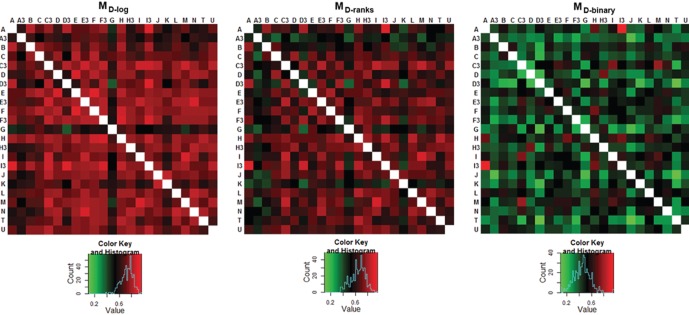
Matrices of cross-reactive antibody binding correlations. Pearson’s correlation (*r*) was calculated between all OspC pairs using antibody binding reactivity values for all 55 sera from patients with LD to populate the matrices shown as heat maps. The matrices computed for log_10_-transformed data (*M_D-log_*), rank transform (*M_D-rank_*) and binary transform (*M_D-binary_*) are shown in the left, middle and right panels, respectively.

Each LD serum sample showed positive antibody binding to more than one OspC type present on the array. The correlation coefficient Pearson’s *r* was used as an indicator of *in vitro* antibody cross-reactive binding between OspC proteins, and the *r* values calculated for each possible pairing populated the cross-reactive antibody binding correlation matrices (*M_D_*) shown in Figure 1. Each heat map presents the matrix calculated using each of the three data transforms (log_10_, rank and binary); the respective numerical *r* values are available in the Table S3.

**Figure 2:**
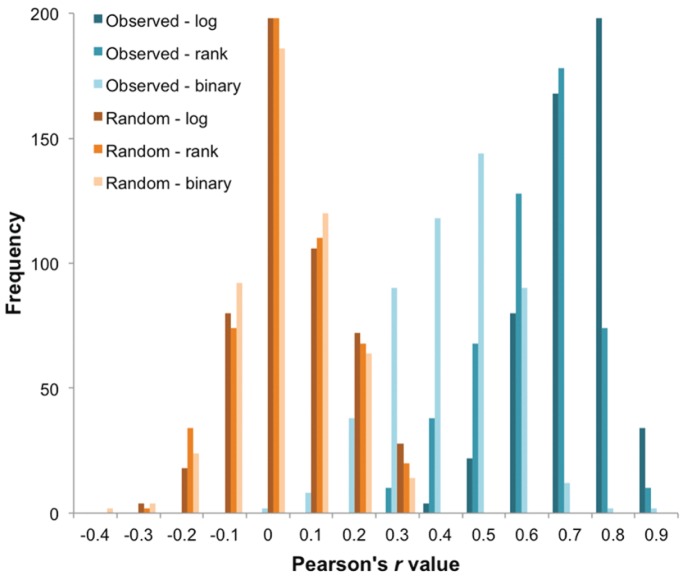
Distribution of Pearson’s *r values* from OspC cross-reactivity matrices. The frequency histogram shows the distribution of *r* values obtained from the OspC cross-reactivity matrices calculated using either the observed (blue bars) or randomized (orange bars) antibody binding profiles and their 3 transform metrics, log_10_, rank and binary.

The 20 most cross-reactive OspC pairs are shown in Table 1, ranked by the average *r* from the three matrices. For sera from patients with LD, the OspC pair A, I3 had the highest cross-reactivity value in all three matrices, followed by the pairs I, M; C3, M; C3, E3; H, I3 and C3, I. The remaining 247 OspC pairs had average *r* values <0.80, with a frequency count for the following ranges: 38 pairs with *r* values between 0.70–0.80, 94 between 0.60–0.70, 73 between 0.50–0.60, 33 between 0.40–0.50, and 9 between 0.30–0.40. The complete list of pairwise OspC cross-reactivity values for the sera sets studied is provided in Table S4. Randomization of the linkages between antibody binding and individual OspC proteins yielded in *r* values with mean of near zero, an indication that the correlations found for the observed values are indicative of the range of antibody binding to OspC proteins resulting from the specificity of immune response and not by chance. Histograms of the correlations from the actual data matrices and the randomized matrices are presented in Figure 2.

**Table 1:** Twenty most cross-reactive OspC types for sera from patients with Lyme disease.

	Pearson’s *r* of Antibody Cross-Reactivity			
OspC Pair	Log_10_	Rank	Binary	Average
A, I3	0.94	0.90	0.89	0.91
I, M	0.88	0.88	0.73	0.83
C3, M	0.86	0.85	0.73	0.81
C3, E3	0.87	0.83	0.72	0.81
H, I3	0.85	0.83	0.73	0.80
C3, I	0.87	0.84	0.68	0.80
D, H3	0.79	0.81	0.75	0.78
D3, I3	0.90	0.87	0.57	0.78
F3, I3	0.89	0.84	0.60	0.78
E, F	0.91	0.82	0.58	0.77
F, H	0.87	0.78	0.64	0.76
A, D3	0.84	0.80	0.64	0.76
E3, I	0.86	0.79	0.63	0.76
F3, H	0.89	0.83	0.53	0.75
H, T	0.84	0.86	0.56	0.75
C3, I3	0.87	0.78	0.59	0.75
H3, T	0.80	0.79	0.65	0.75
H, L	0.81	0.82	0.60	0.74
A, H	0.82	0.76	0.64	0.74
D, E	0.89	0.76	0.57	0.74

### Global Sequence Identity and Cross-reactivity

OspC proteins have both conserved and variable regions of amino acid sequences amongst types. The multiple sequence alignment (*MSA*) of the 23 OspC proteins is presented in Figure S2. The alignments in the *MSA* were used to calculate the sequence identity for each OspC pair, and the resulting values were used to populate the global amino acid sequence identity matrix (*M_S-Global_*). On average, OspC proteins shared 72 (68–76)% in their amino acid sequences for the processed protein, with identity ranging from 90% (OspC types F and I3) to 63% (OspC E and OspC L). The values for the complete *M_S-Global_* matrices are provided in Table S5.

The relationship between the global amino acid sequence identity (*M_S-Global_*) and the cross-reactive antibody binding (*M_D_*) between OspC pairs was calculated, with resulting correlation values for *r*(*M_S-Global_*, *M_D_*) of 0.16, 0.07, and 0.07, for the log_10_, rank and binary transforms, respectively. This result was an indication that the degree of amino acid identity shared between two OspC proteins does not account for most of the observed cross-reactive antibody binding between them.

### OspC Terminal Regions and Antibody Cross-reactivity

**Figure 3:**
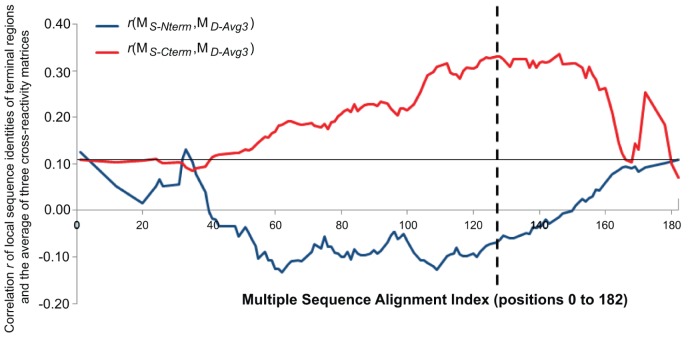
Correlation between local sequence identity of terminal regions and cross-reactivity. The x-axis values correspond to residue positions in the *MSA* index. The y-axis presents the correlation between local sequence identities of terminal regions and the average of three cross-reactivity matrices, i.e. *r*(*M_S-Cterm or Nterm_*, *M_D-avg3_*). The terminal regions used for local sequence identity calculations use all positions before (N-terminal: blue line) or after (C-terminal: red line) the corresponding *MSA* index position. The horizontal black line at y = 0.11 corresponds to the correlation between global sequence identity and the average of three cross-reactivity matrices, i.e. *r*(*M_S-Global_*, *M_D-avg3_*). The dashed vertical line is the breakpoint between the N-terminal and the C-terminal regions at *MSA* index 127.

This analysis was repeated focusing on the N- and C-terminal regions of the OspC molecule, by calculating the correlations between the cross-reactivity matrices and sequence identity matrix using only the terminal regions. Figure 3 presents the correlations between each possible N-terminal or C-terminal region and the average *r* value for the three cross-reactivity matrices (*M_D-avg3_*). The highest correlation value (0.34) was obtained for the C-terminal region beginning with the *MSA* index position 146 (OspC A index position 170). The high correlation associated with the C-terminal portion of the molecule is not sensitive to the precise cut off point used to define the terminal region, as correlation values greater than 0.30 were observed for all C-terminal regions between *MSA* indices 118 and 153.

For the individual cross-reactivity matrices, when the break point was set between *MSA* index positions 127 and 128 (OspC A indices 152 and 153), the correlations calculated using the N-terminal section produced *r*(*M_S-Nterm_*, *M_D_*) values of 0.02, −0.09, and −0.09, for the log_10_, rank and binary transforms, respectively; whereas for correlations using the C-terminal section, the corresponding values were 0.29, 0.31, and 0.28. This was evidence that the C-terminal region of OspC accounts for much of the cross-reactive antibody binding observed.

### Local Sequence Windows and Cross-reactivity

**Figure 4:**
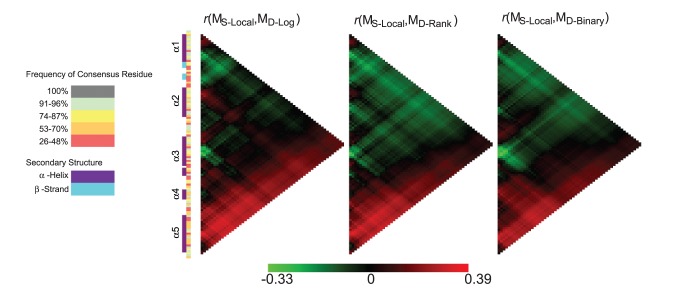
Heat maps of correlation between local sequence identity using subsets of sequentially consecutive polymorphic positions and antibody cross-reactivity. Local sequence regions were systematically defined and correlations were calculated as described in Methods for Sequence Scanning. Each panel shows results from the calculation performed using the 3 transforms: log-transformed, rank and binary, on the left, middle and right panels, respectively. Each row in a heat map corresponds to the center of a sequence window and the columns encompass the results calculated using window sizes varying from 3 to 116 residues (only polymorphic positions are considered in the size). The individual alpha helical structures are indicated α1 through α5 [50]. The green to red gradient bar indicates the range of *r* values observed in the results (min: −0.33; max: 0.39).

The relationship between regions of more divergent sequence across OspC variants and cross-reactivity between pair members was calculated using local sequence identity matrices (*M_S-Local_*) and the cross-reactivity matrices. The highest *r*(*M_S-Local_, M_D_*) values resulted from a window size of 7 residues centered on position 182 in the fifth helix, and were 0.38, 0.39 and 0.30 for the log, rank and binary transforms, respectively. The heat maps summarizing these results are presented in Figure 4; the respective numerical values are available in Table S6.

The sequence window of 7 residues in the restricted *MSA_poly_* corresponded to 10 positions in the full *MSA.* This region spanned residues 179 through 188 of the OspC A index, including 7 polymorphic and 3 conserved positions (L183, K185, A187), and is located in the center of the fifth and last alpha helix, as highlighted in Figure S2. The distance between the C_β_ atoms of residues 179 and 188 is 14.8 Å, as determined by Chimera UCSF [37].

**Figure 5:**
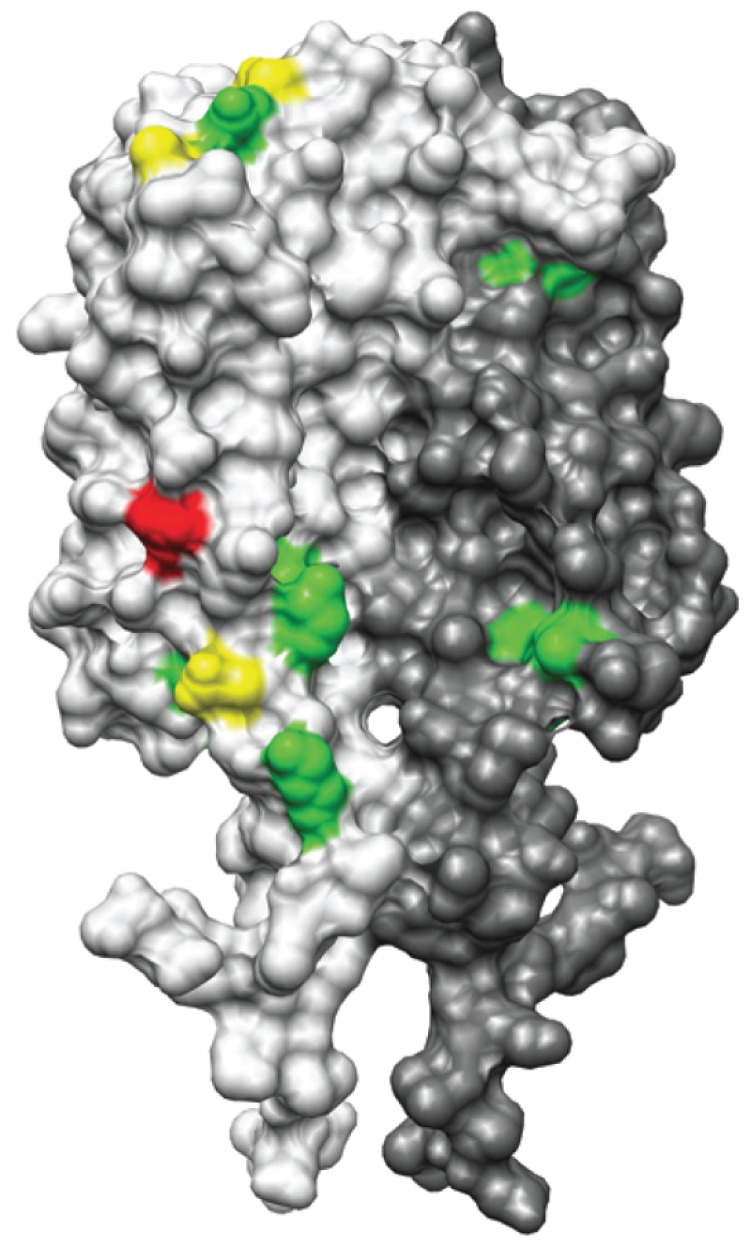
Maximum cross-reactivity regions for individual OspC types. Solvent-accessible surface area representation of the 3D structure model constructed from the *MSA* using UCSF Chimera from the structure of OspC-A (pdb 1GGQ). The chains of OspC dimer are colored light and dark grey. The location of residues of highest correlation (*r* value) with antibody cross-reactivity (*M_D-avg3_*) are colored as follows: highest *r* value in one OspC protein (green); in two OspC proteins (yellow); in five OspC proteins (red).

Until now all calculations considered *r* values using all-versus-all OspC types. However, when the relationship between local sequence identity and antibody cross-reactivity were calculated for an individual OspC type versus all others, the correlation between cross-reactivity and positions 179 through 188 of the fifth helix is more evident. For instance, for OspC type A, the *r* values were 0.75, 0.75, and 0.73 for log, rank and binary transforms, and the corresponding values for OspC type D3 were 0.67, 0.65, and 0.60. The central position of the 7-residue window most correlated with antibody cross-reactivity is shown on the solvent-accessible surface model of the 3D structure constructed from the *MSA* presented in Figure 5. The heat maps in Figure S3 summarize the correlation results for individual residues, highlighting the highest *r* value for each OspC protein in white boxes; the corresponding source values are provided in Table S7.

### Local Structure Clusters and Cross-reactivity

To assess the relationship between cross-reactivity and a subset of residues in close proximity to one another in 3-dimensional space, a sequence identity matrix using only the residue cluster (*M_S-Local3D_*) was generated and correlated with the averaged cross-reactivity matrices. The highest *r*(*M_S-Local3D_*, *M_D-avg3_*) value, 0.38, was found using a sphere with predicted diameter of 8 Å, which encompassed the polymorphic positions 56, 63, 180, 181, 182, 184, 186, and 188 of the OspC A index. Positions 56 and 63 are part of the first helix, while the remaining 6 positions are in the fifth helix. The fifth helix positions are the same as 6 of the 7 positions (the exception being position 179) that were identified by the sequence scanning approach as being most highly correlated with cross-reactivity. All correlation results using sphere sizes 4 to 40Å are available in Table S8.

### Reactivity to OspC I3, a Chimera of Types F and A

**Figure 6:**
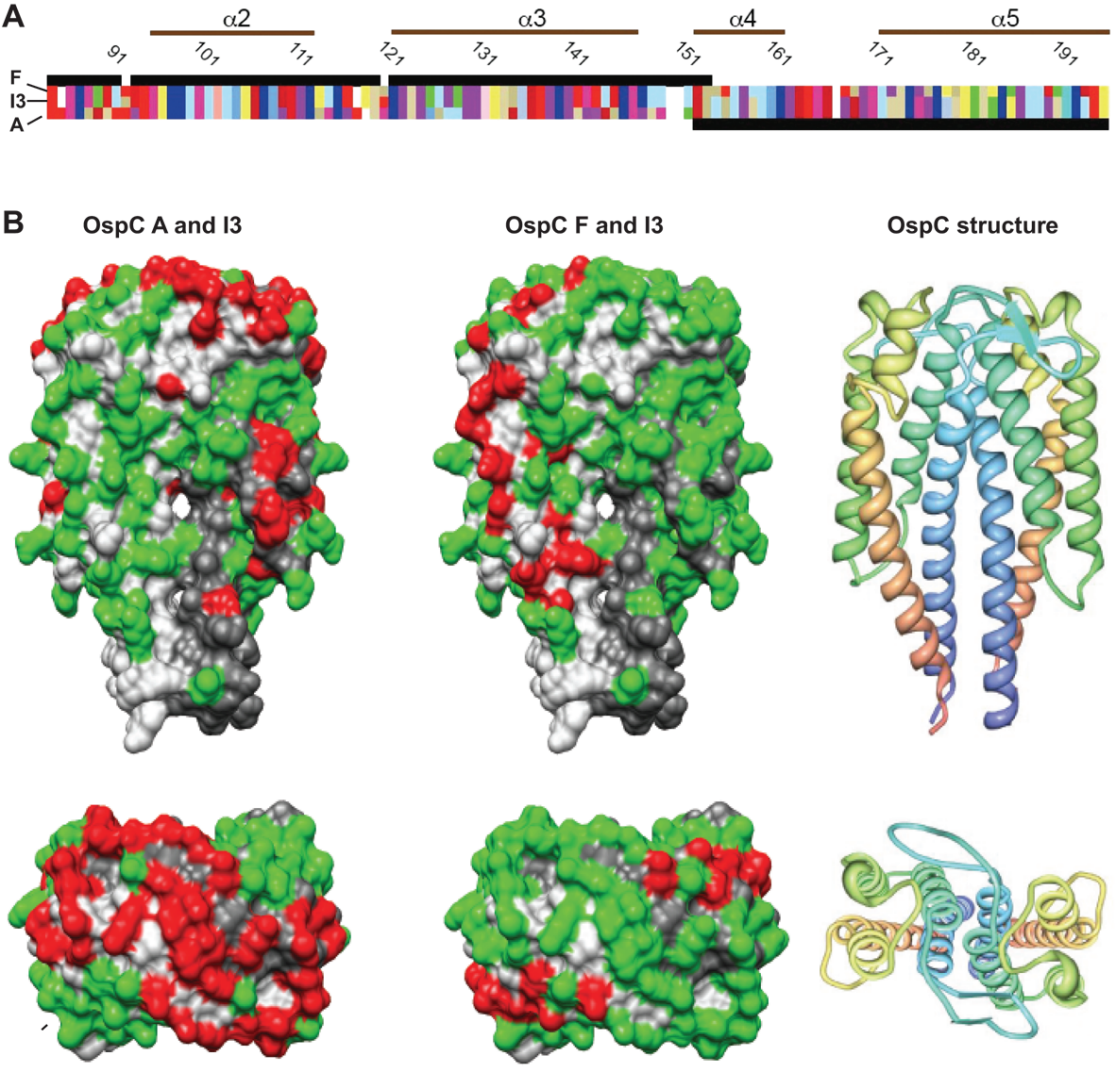
Comparison of conserved and variable residues between OspC pairs A, I3 and F, I3. **Panel A** Partial amino acid sequence alignment of OspC types F (top row), A (bottom row) and the chimeric OspC I3 (middle). Only helices α2 through α5 are shown. Black bars indicate regions of sequence identity between the chimeric OspC I3 to the parental OspC types. Colored blocks represent individual amino acids. **Panel B** Top row: frontal view of the OspC dimer structure; bottom row, view from the top of the structure. The cartoon representation of the OspC dimer is colored from N- to C- terminus in blue to red, respectively. The surface representations show the combined amino acid sequences of pairs OspC A and I3 (left) and OspC F and I3 (center), where polymorphic positions identical within each pair are shown in green and mismatches appear in red. Residues strictly conserved in all 23 OspC proteins appear in light and dark gray.

A naturally occurring chimeric OspC protein provided an opportunity to directly evaluate the importance of the fifth helix for cross-reactivity. OspC I3 comprises helices 1, 2 and 3 of OspC F, and helices 4 and 5 of OspC A [38]. The alignment of the 3 proteins together with the locations of helices 2–5 is shown in Figure 6, panel A. Global amino acid sequence identity between OspC types I3 and F is 90%, while between A and I3 is 80%. Figure 6, panel B shows the pairwise identities according to the 3D structural model, with the sequence matches and mismatches for the OspC pairs indicated by green and red. Only 17 positions differ in the pair F, I3 and all but one occur in the fifth helix. In contrast, the pair A, I3 contains 36 mismatches and all of them are in that portion of OspC proximal to the fifth helix.

**Figure 7:**
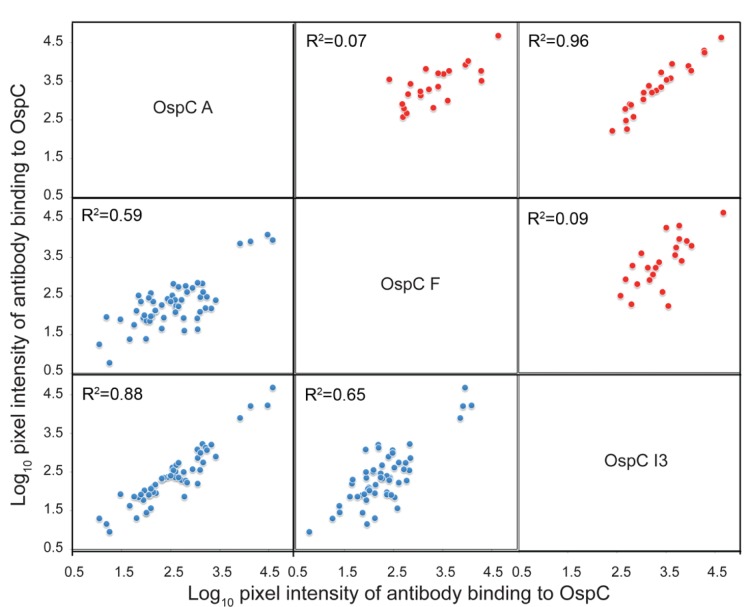
Multivariate analysis of antibody response to OspC types A, F and the chimera I3. Log10-transformed intensity of antibody binding to OspC A, F and I3 by sera from 55 human patients with LD (blue dots) and 23 experimental infections of *P. leucopus* rodents (red dots). Correlation coefficient *R^2^* is shown for each comparison.

For two sets of sera examined, from patients with LD and from *P. leucopus* rodents experimentally infected with *B. burgdorferi,* the pair I3 and A had the highest ranking correlations, with averaged *r* values of 0.91 and 0.95, respectively; while the I3 and F pair was ranked number 118 and 190 out of the 253 possible pairs (Table S4). In Figure 7, the binding of antibodies to the 3 proteins is compared against each other. For both sets of sera the highest coefficients of determination (*R^2^*) were between I3 and A, further evidence of the immunodominance of the fifth helix over global sequence identity.

## Discussion

We described here a computational protocol for analysis of the binding of antibodies to a diverse population of variants of an antigen presented in an array format. The set of proteins are homologous but diverse enough to feature both type-specific epitopes and cross-reactive epitopes. Accurately distinguishing cross-reactive epitopes from type-specific epitopes on the basis of amino acid sequence is a challenging problem. Our analytic approach automatically generates testable hypotheses regarding which specific sets of residues of the full-length protein comprise immunodominant linear or conformational epitopes. As a model system for development of the protocol, we used 23 variants of the polymorphic OspC surface-exposed protein of *B. burgdorferi* and asked whether cross-reactive antibody binding was influenced by the degree of global identity at amino acid level or by specific smaller regions of the protein. To this end, we performed an automated systematic analysis of the relationship between the variation among subsets of positions adjacent in sequence or 3D space and the experimentally observed antibody cross-reactivity produced by a set of sera from individuals with documented LD. We found that cross-reactivity between specific pairs of OspC proteins is determined by sequence identity at positions 179 through 188 of the C-terminal fifth alpha helix, rather than how much global identity is shared between the pair.

A limitation of the study was that the infecting strain (or strains) for patients with LD was not known. If the infecting type was known for each sample, then the quantitative measure of cross-reactivity between pairs of OspC variants could be calculated more directly using only samples infected with specific types. Additionally, the possibility that patients could be infected with more than one strain of *B. burgdorferi* could bias cross-reactivity results; however, multiple strain infections seem to be uncommon in humans [39]. In the context of unknown infecting type we use similarity of antibody binding patterns for OspC pairs over the entire set of samples as a proxy for the ideal quantitative measure. On the other hand, absence of knowledge of the infecting strain is by far the most common circumstance during medical management of LD at present and will likely be for the near-term future, until the means to identify infecting strains become feasible and widely adopted.

Another limitation of the study was the dependence on an assay that measures binding of antibody to purified protein on a matrix and not to an *in situ* protein at the surface of a living bacterium. Presumably all antibodies directed against an OspC protein are not equal in their effector functions, such as direct neutralization or opsonization. Moreover, as previous studies of strain-specificity of protective immunity have indicated [8], [17], [18], only a portion of the anti-OspC antibodies are likely to be functionally active in this regard. On the basis of the established utility of a single OspC protein for immunodiagnostic assays, the study’s array-based assay might not have been expected to tease out subtle type-specific responses. Nevertheless, we showed this was possible in a previous study using this array and experimentally-infected rodents [40], and the differences in reactivity over a range of diverse OspC proteins observed in this study is evidence that even under the conditions where binding by antibodies of little or no functional consequence occurs, we could still detect type-specific binding. This suggests to us that the array-based assays are informative for questions of vaccine or diagnostic design even with a high background of cross-reactivity.

Thus despite the near ubiquitous reactivity to the conserved N-terminal first helix of the OspC protein [40], we determined that the fifth helix is also an immunodominant epitope, as several epitope mapping studies indicated by other approaches [8], [41]–[45]. The independent validation of our results by traditional techniques adds merit to our procedure; however, our high-throughput approach is not a substitute for traditional experimental methods of epitope mapping, but it may be a valuable complement to these.

Although our study represents the broadest effort to determine the immunodominant regions responsible for cross-reactive antibody binding between variants of the OspC protein, the bioinformatics approach described here can be applied in the study of polymorphic immunodominant antigens in other human pathogens. For instance, *Plasmodium falciparum* antigens are promising targets for analysis due to the established links between antigen polymorphism and development of resistance only after exposure to many circulating strains, and the ongoing large-scale effort to investigate immune responses of individuals and populations suffering from malaria [46], [47].

The two types of data that are necessary for performing the cross-reactivity analysis for a set of variants are: (1) quantitative measurements of antibody binding to each variant for multiple patient samples and (2) a multiple sequence alignment of the corresponding sequence variants. For structure scanning, a 3D structure model is also required. The methods for performing the systematic terminal region scanning, sequence scanning, and structure scanning are implemented in a suite of Perl scripts. These scripts, as well as sample input and output files from the OspC project, are publicly available at http://download.igb.uci.edu#ospc.

## Materials and Methods

### Sera

The 55 sera from adult patients with different stages of LD and the 25 sera from naïve adults were described in detail previously [36]. In brief, 27 patient and 5 control sera were provided by the Centers for Disease Control and Prevention, Fort Collins, CO, and 28 patient and 20 control sera were provided by Allen Steere, Harvard University. Sera from the 23 *P. leucopus* experimentally-infected with *B. burgdorferi* isolates HB19, Sh-2-82, IDS, TBO2, WQR and 27577 and the 7 control sera were described in detail in [40]. Briefly, adult female pathogen-free, closed-colony outbred *P. leucopus* (LL stock; Peromyscus Genetic Stock Center, University of South Carolina) were inoculated intraperitoneally with fresh CB-17 SCID mice plasma containing host-adapted *B. burgdorferi* cells. Animals were terminally exsanguinated 5 weeks post-infection. Samples were kept frozen at −80°C until use.

### Ethics Statement

Sera from human donors were originally collected for other studies for which informed consent had been obtained; patient identifier information had been removed. Rodent serum samples were obtained as described in [40], and the study was carried out in strict accordance with the recommendations in the Guide for the Care and Use of Laboratory Animals of the National Institutes of Health. The protocol was approved by the Institutional Animal Care and Use Committee of the University of California Irvine (IACUC protocol 1999–2080).

### Preparation, Construction and Probing of the OspC Protein Microarray

#### Amplification and cloning of ospC alleles

Table S9 provides the sources, geographic origins, accession numbers and references for the *ospC* alleles cloned. Table S10 lists the name and nucleotide sequence of primers utilized to amplify these genes. *ospC* ORFs coding for protein sequence without signal peptide were cloned into pXT7 expression vector containing an amino-terminal 10X-Histidine fusion tag, using the *in vivo* recombination cloning method [35]. For details regarding PCR reactions and cloning methods, please refer to Text S1.

#### OspC protein expression and purification

BL21(DE3)*pLysS E. coli* cells transformed with pXT7-*ospC* plasmids were cultured in Terrific Broth (MP Biomedicals, Solon, OH) supplemented with kanamycin until reaching OD_600_ 0.4–0.6. Recombinant protein expression was inducted with IPTG (RPI, Mt. Prospect, IL) and further incubation for additional 4 hours. Cells were harvested and the supernadant containing His-tagged OspC fusion protein was incubated with Ni-coupled magnetic beads (MagneHis kit, Promega, Madison, WI) for protein purification. Recombinant protein purity was estimated to be 80–90% by densitometry of Coomassie Blue-stained protein bands on sodium dodecyl sulfate-polyacrylamide (SDS-PAGE) gels, and concentration was determined by BCA Protein Assay kit (Pierce, Rockford, IL). Purified OspC protein samples were aliquoted and stored at −80°C until use. For details on recombinant protein purification, refer to Text S1.

#### OspC protein array printing

Purified recombinant-OspC proteins were printed on nitrocellulose-coated glass FAST slides (Whatman, Piscataway, NJ) using an Omnigrid 100 apparatus (Digilab, Holliston, MA), in duplicate spots and in approximately 10 pg and 30 pg of protein per spot. Protein storage buffer alone was printed on the array to serve as a background signal control.

#### OspC protein microarray probing

Serum samples were diluted 1∶200 (LD patient sera) or 1∶100 (*P. leucopus*) in Protein Array Blocking (PAB) buffer (Whatman Inc, Sanford, ME) supplemented with 10% (vol/vol) DH5α *E. coli* lysate (MCLAB, San Francisco, CA). Incubation and washing procedures are described in Text S1. Cy3-conjugated secondary antibody, goat anti-human IgG heavy and light chain or goat anti-*Peromyscus leucopus* IgG heavy and light chain (KPL, Gaithersburg, MD) were used to detect sera antibody binding to OspC proteins. Probed array slides were scanned in a Perkin Elmer ScanArray Express HT and output RGB TIFF files were quantitated using ProScanArray Express software (Perkin Elmer, Waltham, MA) with spot-specific background correction. The array data is deposited in NCBI’s Gene Expression Omnibus [48] and is accessible through GEO Series accession number GSE45996 (http://www.ncbi.nlm.nih.gov/geo/query/acc.cgi?acc=GSE45996).

### Protein Microarray Data Analysis

#### Primary analysis

Inclusion criterion for cross-reactivity analysis was a minimum reactivity corresponding to a z score of 2 to at least one OspC protein. For analysis of antibody binding to OspC proteins on the microarray the following steps were taken: (i) raw values of antibody binding measured as the mean pixel intensity of spots of printed protein were log_10_-transformed; raw values less than 1.0 were set to 0; (ii) the mean, standard deviation, 95% confidence intervals and z-scores of antibody binding intensity to each OspC type were calculated for the LD patient, *P. leucopus* and respective control sera groups.

#### Cross-reactivity analysis

For a given OspC type *x* the row of all individual raw reactivity values is denoted by *D_x_* (e.g., *D_A_* contains reactivity to OspC type A). For a given pair of OspC variants the Pearson’s correlation *r* between the corresponding rows of serum reactivity values was used as the quantitative measure of cross-reactive antibody binding. The correlations were calculated using three forms of transformed data: log_10_ (reactivity values were log_10_-transformed); rank (for each OspC type, ranks sera from lowest (1) to highest reactivity value (55 for human sera or 23 for *P. leucopus* sera)); and binary (values above the global median were set to 1 and values below were set to 0). For each of the transforms, all possible pairwise correlations between two OspC types were calculated and saved in the corresponding antibody cross-reactivity matrices: *M_D-log_*, *M_D-rank,_ M_D-binary_*_._ Please refer to Abbreviations section in Text S1 for further explanation.

### OspC Sequence and Structure Analysis

#### Structure-based multiple sequence alignment

A draft multiple sequence alignment (*MSA*) of the OspC proteins was assembled using PSI-BLAST [49] and then manually adjusted to accommodate insertions and deletions. The *MSA* comprises residues 31 to 206 of OspC A using the indexes of Kumaran *et al*. [50], denoted as OspC A Index. The modeled consensus sequence consisted of 183 residues; whereas the individual sequences ranged from 175 to 179 residues over the aligned positions, considering gaps. The pairwise alignments from the *MSA* were used for all global and local amino acid sequence identity calculations and the aligned gaps between OspC pairs were counted as identities.

#### 3D structure model and inter-residue distances

A 3-dimensional model of the *MSA* was constructed using Modeller 9.1 [51] with the structures of OspC A (pdb 1GGQ), OspC E (pdb 1G5Z) and OspC I (pdb 1F1M) [50], [52] as templates, and the consensus sequence (ignoring gaps) as the target sequence to be modeled. Distances between C_β_ atoms (C_α_ atoms for glycine) in the model were used to define inter-residue distances for determining spatial clusters of residues.

### Systematic Comparison of Local Sequence Identity Matrices and Cross-reactivity Matrices

The three cross-reactivity correlation matrices (M_D-log_, M_D-rank_, and M_D-binary_) were compared to the global sequence identity matrix (M_S-Global_) and the similarity was summarized by the Pearson’s correlation of the paired matrices: r(M_S-Global_, M_D-log_), r(M_S-Global_, M_D-rank_), and r(M_S-Global_, M_D-binary_). Similarly, local sequence identity matrices (M_S-Local_) were calculated systematically using subsets of positions that were adjacent in sequence or in 3-dimensional structure (M_S-Local3D_) and compared to the cross-reactivity matrices.

#### Sequence scanning

Sequence windows were defined using only the 116 polymorphic positions in *MSA_poly_*. Each position in *MSA_poly_* was treated as the center of a window encompassing 3 consecutive polymorphic positions and the correlation between the corresponding local sequence identity matrix and the cross-reactivity matrices were calculated, i.e. *r*(*M_S-Local_*, *M_D-log_*), *r*(*M_S-Local_*, *M_D-rank_*), and *r*(*M_S-Local_*, *M_D-binary_*). The process was repeated for all odd window sizes from 3 to 115 positions. For individual types (e.g. OspC A vs. the other 22 types) the relationship between cross-reactivity and local sequence identity was calculated restricted to corresponding rows of the matrices (e.g. *r*(*M_S-Local_*[OspC A], *M_D-log_*[OspC A]) and a fixed window size of 7 polymorphic positions.

#### Structure scanning

Structural clusters of residues were defined using only the polymorphic positions in *MSA_poly_*. Each position was used as the central residue for defining a cluster of residues in 3-dimensional space where membership in the cluster was defined by proximity of less than 4 Å to the central residue. The correlations between the corresponding sequence identity matrix calculated using the residue cluster (*M_S-Local3D_*) and the cross-reactivity matrices were calculated, using distance thresholds of 4 to 40 Å in 4 Å increments.

## Supporting Information

Figure S1 **Box plot of antibody binding to OspC proteins by sera from patients with LD and healthy controls.** The intensity of antibody binding to each OspC protein on the microarray is shown for patient (blue) and control (white) sera. Each box indicates the first and third quartiles, and the line inside the box is the median. The 1.5x interquartile range is indicated by the vertical line (whiskers) bisecting the box, and values outside this range are indicated by dots.
(EPS)

Figure S2 **Multiple sequence alignment (** ***MSA*** **) of conserved and variable positions among 23 OspC types.** Strictly conserved positions are shown in gray; positions with variability are colored according to the number of residues that do not match the consensus residue at a given position. Color scheme is presented in the image key. The portion of the fifth C-terminal helix most correlated with cross-reactivity is outlined with a red box. OspC A index, position of residue in the crystal structure of OspC A [50]; *MSA* index, residue position relative to *MSA*; Secondary structure, as described for OspC A, showing alpha helices α1 through α5 [50]; Consensus, most frequent amino acid at each position amongst the 23 OspC proteins in the alignment.
(EPS)

Figure S3 **Heat maps of correlation between local sequence identity and antibody cross-reactivity for individual OspC types.** All results were calculated using a window size of 7 positions (excluding conserved positions). The green to red gradient bar indicates the range of *r* values observed (min: −0.72; max: 0.77). White boxes around individual residues indicate the highest *r* value for each OspC type. Each panel shows results from the calculation performed using the 3 transforms: log_10_-transformed, rank and binary, on the left, middle and right panels, respectively.
(EPS)

Figure S4 **Purified OspC proteins.** Coomassie-blue stained SDS-PAGE gels showing the migration of affinity-purified recombinant OspC proteins expressed and purified for this study. The OspC type, designated alphanumerically, is shown on the top of the figure. The migrations of molecular weight markers, in kilodaltons, are shown on the left-most column.
(EPS)

Table S1 **Antibody binding by sera from patients with LD and controls to conserved** ***B. burgdorferi*** **proteins and B31 strain whole cell lysate.**
(DOC)

Table S2 **Individual serum sample information and pixel intensities for antibody binding to OspC proteins on the microarray.**
(XLS)

Table S3 **Pearson’s correlation** ***r*** **values of antibody cross-reactivity between OspC proteins using log_10_, rank, binary data transforms and their average (** ***M_D_*** **).**
(XLS)

Table S4 **Ranking of pairwise cross-reactivity** ***r*** **values between OspC proteins, calculated for sera from human patients with LD and** ***P. leucopus*** **rodents infected with** ***B. burgdorferi*** **.**
(XLS)

Table S5 **Pairwise global amino acid sequence identity matrix between 23 OspC proteins (** ***M_S-Global_*** **), in percentage.**
(XLS)

Table S6 **Pearson’s** ***r*** **values of correlation between local sequence identity matrix (** ***M_S-Local_*** **)** **using polymorphic positions and the antibody cross-reactivity matrices (** ***M_D-log_, M_D-rank_, M_D-binary_*** **).**
(XLS)

Table S7 **Pearson’s** ***r*** **values between local sequence identity and antibody cross-reactivity matrices for individual OspC types.**
(XLS)

Table S8 **Pearson’s** ***r*** **values between antibody cross-reactivity matrices and local sequence identity in 3 dimentional space (** ***M_S-Local3D_*** **), using distance thresholds of 4 to 40 Å.**
(XLS)

Table S9 **Sources of genomic template for** ***ospC*** **allele cloning.**
(DOC)

Table S10 **Nucleotide sequences of PCR primers used for cloning and sequencing of** ***ospC*** **alleles.**
(DOC)

Text S1 **Detailed information on Materials and Methods.**
(DOC)

## References

[1] Kringelum JV, Nielsen M, Padkjaer SB, Lund O (2013) Structural analysis of B-cell epitopes in antibody:protein complexes. Mol Immunol 53(1–2): 24–34. 22784991 10.1016/j.molimm.2012.06.001 PMC3461403

[2] Korber B, LaBute M, Yusim K (2006) Immunoinformatics comes of age. PLoS Comput Biol 2(6): e71. 16846250 10.1371/journal.pcbi.0020071 PMC1484584

[3] Chen P, Rayner S, Hu KH (2011) Advances of bioinformatics tools applied in virus epitopes prediction. Virol Sin 26(1): 1–7. 21331885 10.1007/s12250-011-3159-4 PMC7090880

[4] Yang X, Yu X (2009) An introduction to epitope prediction methods and software. Rev Med Virol 19(2): 77–96. 19101924 10.1002/rmv.602

[5] Kraiczy P, Hunfeld KP, Peters S, Wurzner R, Ackert G, et al (2000) Borreliacidal activity of early Lyme disease sera against complement-resistant Borrelia afzelii FEM1 wild-type and an OspC-lacking FEM1 variant. J Med Microbiol 49(10): 917–928. 11023189 10.1099/0022-1317-49-10-917

[6] Montgomery RR, Malawista SE, Feen KJ, Bockenstedt LK (1996) Direct demonstration of antigenic substitution of Borrelia burgdorferi ex vivo: exploration of the paradox of the early immune response to outer surface proteins A and C in Lyme disease. J Exp Med 183(1): 261–269. 8551229 10.1084/jem.183.1.261 PMC2192432

[7] Rousselle JC, Callister SM, Schell RF, Lovrich SD, Jobe DA, et al (1998) Borreliacidal antibody production against outer surface protein C of Borrelia burgdorferi. J Infect Dis 178(3): 733–741. 9728542 10.1086/515382

[8] Jobe DA, Lovrich SD, Schell RF, Callister SM (2003) C-terminal region of outer surface protein C binds borreliacidal antibodies in sera from patients with Lyme disease. Clin Diagn Lab Immunol 10(4): 573–578. 12853388 10.1128/CDLI.10.4.573-578.2003 PMC164245

[9] Barbour AG, Travinsky B (2010) Evolution and Distribution of the ospC Gene, a Transferable Serotype Determinant of Borrelia burgdorferi. MBio, 1(4). 10.1128/mBio.00153-10 PMC2945197 20877579

[10] Wilske B, Preac-Mursic V, Jauris S, Hofmann A, Pradel I, et al (1993) Immunological and molecular polymorphisms of OspC, an immunodominant major outer surface protein of Borrelia burgdorferi. Infect Immun 61(5): 2182–2191. 8478108 10.1128/iai.61.5.2182-2191.1993 PMC280819

[11] Zhong W, Stehle T, Museteanu C, Siebers A, Gern L, et al (1997) Therapeutic passive vaccination against chronic Lyme disease in mice. Proc Natl Acad Sci U S A 94(23): 12533–12538. 9356484 10.1073/pnas.94.23.12533 PMC25028

[12] Probert WS, LeFebvre RB (1994) Protection of C3H/HeN mice from challenge with Borrelia burgdorferi through active immunization with OspA, OspB, or OspC, but not with OspD or the 83-kilodalton antigen. Infect Immun 62(5): 1920–1926. 8168958 10.1128/iai.62.5.1920-1926.1994 PMC186442

[13] Preac-Mursic V, Wilske B, Patsouris E, Jauris S, Will G, et al (1992) Active immunization with pC protein of Borrelia burgdorferi protects gerbils against B. burgdorferi infection. Infection 20(6): 342–349. 1293055 10.1007/BF01710681

[14] Mbow ML, Gilmore RD Jr, Titus RG (1999) An OspC-specific monoclonal antibody passively protects mice from tick-transmitted infection by Borrelia burgdorferi B31. Infect Immun 67(10): 5470–5472. 10496931 10.1128/iai.67.10.5470-5472.1999 PMC96906

[15] Gilmore RD Jr, Kappel KJ, Dolan MC, Burkot TR, Johnson BJ (1996) Outer surface protein C (OspC), but not P39, is a protective immunogen against a tick-transmitted Borrelia burgdorferi challenge: evidence for a conformational protective epitope in OspC. Infect Immun 64(6): 2234–2239. 8675332 10.1128/iai.64.6.2234-2239.1996 PMC174061

[16] Bockenstedt LK, Hodzic E, Feng S, Bourrel KW, de Silva A, et al (1997) Borrelia burgdorferi strain-specific Osp C-mediated immunity in mice. Infect Immun 65(11): 4661–4667. 9353047 10.1128/iai.65.11.4661-4667.1997 PMC175668

[17] Brown EL, Kim JH, Reisenbichler ES, Hook M (2005) Multicomponent Lyme vaccine: three is not a crowd. Vaccine 23(28): 3687–3696. 15882529 10.1016/j.vaccine.2005.02.006

[18] Probert WS, Crawford M, Cadiz RB, LeFebvre RB (1997) Immunization with outer surface protein (Osp) A, but not OspC, provides cross-protection of mice challenged with North American isolates of Borrelia burgdorferi. J Infect Dis 175(2): 400–405. 9203661 10.1093/infdis/175.2.400

[19] Centers for Disease Prevention and Control (1995) Recommendations for test performance and interpretation from the Second National Conference on Serologic Diagnosis of Lyme Disease. MMWR Morb Mortal Wkly Rep 44(31): 590–591. 7623762

[20] Dressler F, Whalen JA, Reinhardt BN, Steere AC (1993) Western blotting in the serodiagnosis of Lyme disease. J Infect Dis 167(2): 392–400. 8380611 10.1093/infdis/167.2.392

[21] Ivanova L, Christova I, Neves V, Aroso M, Meirelles L, et al (2009) Comprehensive seroprofiling of sixteen B. burgdorferi OspC: Implications for Lyme disease diagnostics design. Clin Immunol 132(3): 393–400. 19576856 10.1016/j.clim.2009.05.017 PMC2752154

[22] Bacon RM, Biggerstaff BJ, Schriefer ME, Gilmore RD Jr, Philipp MT, et al (2003) Serodiagnosis of Lyme disease by kinetic enzyme-linked immunosorbent assay using recombinant VlsE1 or peptide antigens of Borrelia burgdorferi compared with 2-tiered testing using whole-cell lysates. J Infect Dis 187(8): 1187–1199. 12695997 10.1086/374395 PMC7109709

[23] Porwancher RB, Hagerty CG, Fan J, Landsberg L, Johnson BJ, et al (2011) Multiplex immunoassay for Lyme disease using VlsE1-IgG and pepC10-IgM antibodies: improving test performance through bioinformatics. Clin Vaccine Immunol 18(5): 851–859. 21367982 10.1128/CVI.00409-10 PMC3122529

[24] Du W, Ma X, Nyman D, Povlsen K, Akguen N, et al (2007) Antigen biochips verify and extend the scope of antibody detection in Lyme borreliosis. Diagn Microbiol Infect Dis 59(4): 355–363. 17888607 10.1016/j.diagmicrobio.2007.06.006

[25] Travinsky B, Bunikis J, Barbour AG (2010) Geographic differences in genetic locus linkages for Borrelia burgdorferi. Emerg Infect Dis 16(7): 1147–1150. 20587192 10.3201/eid1607.091452 PMC3321895

[26] Wormser GP, Brisson D, Liveris D, Hanincova K, Sandigursky S, et al (2008) Borrelia burgdorferi genotype predicts the capacity for hematogenous dissemination during early Lyme disease. J Infect Dis 198(9): 1358–1364. 18781866 10.1086/592279 PMC2776734

[27] Wormser GP, Liveris D, Nowakowski J, Nadelman RB, Cavaliere LF, et al (1999) Association of specific subtypes of Borrelia burgdorferi with hematogenous dissemination in early Lyme disease. J Infect Dis 180(3): 720–725. 10438360 10.1086/314922

[28] Dykhuizen DE, Brisson D, Sandigursky S, Wormser GP, Nowakowski J, et al (2008) The propensity of different Borrelia burgdorferi sensu stricto genotypes to cause disseminated infections in humans. Am J Trop Med Hyg 78(5): 806–810. 18458317 PMC2387051

[29] Seinost G, Dykhuizen DE, Dattwyler RJ, Golde WT, Dunn JJ, et al (1999) Four clones of Borrelia burgdorferi sensu stricto cause invasive infection in humans. Infect Immun 67(7): 3518–3524. 10377134 10.1128/iai.67.7.3518-3524.1999 PMC116539

[30] Wang G, Ojaimi C, Iyer R, Saksenberg V, McClain SA, et al (2001) Impact of genotypic variation of Borrelia burgdorferi sensu stricto on kinetics of dissemination and severity of disease in C3H/HeJ mice. Infect Immun 69(7): 4303–4312. 11401967 10.1128/IAI.69.7.4303-4312.2001 PMC98500

[31] Wang G, Ojaimi C, Wu H, Saksenberg V, Iyer R, et al (2002) Disease severity in a murine model of lyme borreliosis is associated with the genotype of the infecting Borrelia burgdorferi sensu stricto strain. J Infect Dis 186(6): 782–791. 12198612 10.1086/343043 PMC2773673

[32] Doolan DL, Mu Y, Unal B, Sundaresh S, Hirst S, et al (2008) Profiling humoral immune responses to P. falciparum infection with protein microarrays. Proteomics 8(22): 4680–4694. 18937256 10.1002/pmic.200800194 PMC3021802

[33] Felgner PL, Kayala MA, Vigil A, Burk C, Nakajima-Sasaki R, et al (2009) A Burkholderia pseudomallei protein microarray reveals serodiagnostic and cross-reactive antigens. Proc Natl Acad Sci U S A 106(32): 13499–13504. 19666533 10.1073/pnas.0812080106 PMC2717108

[34] Davies DH, Wyatt LS, Newman FK, Earl PL, Chun S, et al (2008) Antibody profiling by proteome microarray reveals the immunogenicity of the attenuated smallpox vaccine modified vaccinia virus ankara is comparable to that of Dryvax. J Virol 82(2): 652–663. 17977963 10.1128/JVI.01706-07 PMC2224576

[35] Davies DH, Liang X, Hernandez JE, Randall A, Hirst S, et al (2005) Profiling the humoral immune response to infection by using proteome microarrays: high-throughput vaccine and diagnostic antigen discovery. Proc Natl Acad Sci U S A 102(3): 547–552. 15647345 10.1073/pnas.0408782102 PMC545576

[36] Barbour AG, Jasinskas A, Kayala MA, Davies DH, Steere AC, et al (2008) A genome-wide proteome array reveals a limited set of immunogens in natural infections of humans and white-footed mice with Borrelia burgdorferi. Infect Immun 76(8): 3374–3389. 18474646 10.1128/IAI.00048-08 PMC2493225

[37] Pettersen EF, Goddard TD, Huang CC, Couch GS, Greenblatt DM, et al (2004) UCSF Chimera–a visualization system for exploratory research and analysis. J Comput Chem 25(13): 1605–1612. 15264254 10.1002/jcc.20084

[38] Girard YA, Travinsky B, Schotthoefer A, Fedorova N, Eisen RJ, et al (2009) Population structure of the lyme borreliosis spirochete Borrelia burgdorferi in the western black-legged tick (Ixodes pacificus) in Northern California. Appl Environ Microbiol 75(22): 7243–7252. 19783741 10.1128/AEM.01704-09 PMC2786521

[39] Seinost G, Golde WT, Berger BW, Dunn JJ, Qiu D, et al (1999) Infection with multiple strains of Borrelia burgdorferi sensu stricto in patients with Lyme disease. Arch Dermatol 135(11): 1329–1333. 10566830 10.1001/archderm.135.11.1329

[40] Baum E, Hue F, Barbour A (2012) Experimental infections of the reservoir species Peromyscus leucopus with diverse strains of Borrelia burgdorferi, a Lyme disease agent. mBio 3(6): e00434–00412. 23221801 10.1128/mBio.00434-12 PMC3517863

[41] Earnhart CG, Buckles EL, Dumler JS, Marconi RT (2005) Demonstration of OspC type diversity in invasive human lyme disease isolates and identification of previously uncharacterized epitopes that define the specificity of the OspC murine antibody response. Infect Immun 73(12): 7869–7877. 16299277 10.1128/IAI.73.12.7869-7877.2005 PMC1307023

[42] Earnhart CG, Buckles EL, Marconi RT (2007) Development of an OspC-based tetravalent, recombinant, chimeric vaccinogen that elicits bactericidal antibody against diverse Lyme disease spirochete strains. Vaccine 25(3): 466–480. 16996663 10.1016/j.vaccine.2006.07.052

[43] Mathiesen MJ, Holm A, Christiansen M, Blom J, Hansen K, et al (1998) The dominant epitope of Borrelia garinii outer surface protein C recognized by sera from patients with neuroborreliosis has a surface-exposed conserved structural motif. Infect Immun 66(9): 4073–4079. 9712750 10.1128/iai.66.9.4073-4079.1998 PMC108488

[44] Mathiesen MJ, Christiansen M, Hansen K, Holm A, Asbrink E, et al (1998) Peptide-based OspC enzyme-linked immunosorbent assay for serodiagnosis of Lyme borreliosis. J Clin Microbiol 36(12): 3474–3479. 9817857 10.1128/jcm.36.12.3474-3479.1998 PMC105224

[45] Lovrich SD, Jobe DA, Schell RF, Callister SM (2005) Borreliacidal OspC antibodies specific for a highly conserved epitope are immunodominant in human lyme disease and do not occur in mice or hamsters. Clin Diagn Lab Immunol 12(6): 746–751. 15939749 10.1128/CDLI.12.6.746-751.2005 PMC1151971

[46] Drew DR, Hodder AN, Wilson DW, Foley M, Mueller I, et al (2012) Defining the antigenic diversity of Plasmodium falciparum apical membrane antigen 1 and the requirements for a multi-allele vaccine against malaria. PLoS One 7(12): e51023. 23227229 10.1371/journal.pone.0051023 PMC3515520

[47] Blomqvist K, Albrecht L, Quintana Mdel P, Angeletti D, Joannin N, et al (2013) A Sequence in Subdomain 2 of DBL1alpha of Plasmodium falciparum Erythrocyte Membrane Protein 1 Induces Strain Transcending Antibodies. PLoS One 8(1): e52679. 23335956 10.1371/journal.pone.0052679 PMC3546040

[48] Edgar R, Domrachev M, Lash AE (2002) Gene Expression Omnibus: NCBI gene expression and hybridization array data repository. Nucleic Acids Res 30(1): 207–210. 11752295 10.1093/nar/30.1.207 PMC99122

[49] Altschul SF, Madden TL, Schaffer AA, Zhang J, Zhang Z, et al (1997) Gapped BLAST and PSI-BLAST: a new generation of protein database search programs. Nucleic Acids Res 25(17): 3389–3402. 9254694 10.1093/nar/25.17.3389 PMC146917

[50] Kumaran D, Eswaramoorthy S, Luft BJ, Koide S, Dunn JJ, et al (2001) Crystal structure of outer surface protein C (OspC) from the Lyme disease spirochete, Borrelia burgdorferi. Embo J 20(5): 971–978. 11230121 10.1093/emboj/20.5.971 PMC145497

[51] Fiser A, Sali A: Modeller: Generation and Refinement of Homology-Based Protein Structure Models. In: Methods in Enzymology. vol. Volume 374: Academic Press 2003: 461–491. 10.1016/S0076-6879(03)74020-8 14696385

[52] Eicken C, Sharma V, Klabunde T, Owens RT, Pikas DS, et al (2001) Crystal structure of Lyme disease antigen outer surface protein C from Borrelia burgdorferi. J Biol Chem 276(13): 10010–10015. 11139584 10.1074/jbc.M010062200

