# Supplementary material for: Inferring Epitopes of a Polymorphic Antigen Amidst Broadly Cross-Reactive Antibodies Using Protein Microarrays: A Study of OspC Proteins of Borrelia burgdorferi
Source: PLoS One. 2013 Jun 24;8(6):e67445. doi: 10.1371/journal.pone.0067445 (PMC3691210; doi:10.1371/journal.pone.0067445)
Supplement: Table S1 — Antibody binding by sera from patients with LD and controls to conserved B. burgdorferi proteins and B31 strain whole cell lysate. (DOC) [file pone.0067445.s005.doc]

| ***B. burgdorferi* Antigen** | **Antibody Binding Pixel Intensity***^a^* | | **T-test p** |
| --- | --- | --- | --- |
|  | **Controls** | **LD sera** |  |
| B31 whole cell lysate | 2,818 (1,995 - 3,981) | 11,748 (10,000 -13,803) | 4.00E-009 |
| BBK07 | 177 (134 - 229) | 707 (537 - 912) | 1.00E-010 |
| BBK12 | 158 (109 - 234) | 426 (323 - 575) | 9.00E-005 |
| BBK19 | 109 (67 - 173) | 2,398 (1,288 - 4,466) | 8.00E-012 |
| BdrT | 162 (128 - 204) | 707 (478 -1,047) | 4.00E-009 |
| BmpA | 316 (229 - 436) | 5,623 (3,890 - 8,317) | 1.00E-018 |
| DbpB | 114 (91 - 147) | 1,905 (1,122 - 3,235) | 8.00E-015 |
| FlaB | 416 (251 - 691) | 5,011 (3,467 - 7,244) | 1.00E-010 |
| FlgE | 162 (125 - 204) | 489 (371 - 645) | 2.00E-008 |
| FlgG | 158 (120 - 208) | 660 (478 - 912) | 2.00E-009 |
| VlsE | 977 (776 - 1,230) | 2,818 (2,041 - 3,890) | 5.00E-007 |
| *^a^* Mean (95% confidence interval) of raw pixel intensity | | | |
