# Supplementary material for: Inferring Epitopes of a Polymorphic Antigen Amidst Broadly Cross-Reactive Antibodies Using Protein Microarrays: A Study of OspC Proteins of Borrelia burgdorferi
Source: PLoS One. 2013 Jun 24;8(6):e67445. doi: 10.1371/journal.pone.0067445 (PMC3691210; doi:10.1371/journal.pone.0067445)
Supplement: Table S9 — Sources of genomic template for ospC allele cloning. (DOC) [file pone.0067445.s013.doc]

| ***ospC* Allele** | ***B. burgdorferi* Source** | **Location** | **GenBank Accession No.** | **Reference** |
| --- | --- | --- | --- | --- |
| A | Strain B31 (ATCC 35210) | NY | X69596 | [46] |
| A3 | *Ixodes scapularis* | IL | EF592541 | [20] |
| B | *Ixodes scapularis* | NY | CP001422 | - |
| C | *Ixodes scapularis* | MD | DQ437462 | [47] |
| C3 | *Ixodes scapularis* | MN | EF592543 | [20] |
| D | Isolate from dog OUQ | RI | CP001484 | - |
| D3 | *Ixodes scapularis* | WI | EF592544 | [20] |
| E | Isolate from dog XFR | RI | AY275221 | [48] |
| E3 | *Ixodes scapularis* | WI | EF592545 | [20] |
| F | Isolate from dog PRQ | RI | L42896 | [49] |
| F3 | *Ixodes scapularis* | WI | EF592547 | [20] |
| G | Isolate from dog QWQ | RI | AY275223 | [48] |
| H | *Ixodes scapularis* | MN | CP001271 | - |
| H3 | Strain HPN6 | CA | FJ932733 | [33] |
| I | Strain HB19 | CT | AY275219 | [48] |
| I3 | Strain HWT44 | CA | FJ932734 | [33] |
| J | *Ixodes scapularis* | WI | CP001535 | - |
| K | Strain Sh2-2-82 | NY | AY275214 | [50] |
| L | *Ixodes scapularis* | MN | EU375832 | [51] |
| M | *Ixodes scapularis* | MI | CP001550 | - |
| N | *Ixodes scapularis* | NY | EU377775 | [51] |
| T | Isolate 1 from dog WQR | RI | AY275222 | [48] |
| U | Isolate 2 from dog WQR | RI | CP001493 | - |
