# Supplementary material for: Inferring Epitopes of a Polymorphic Antigen Amidst Broadly Cross-Reactive Antibodies Using Protein Microarrays: A Study of OspC Proteins of Borrelia burgdorferi
Source: PLoS One. 2013 Jun 24;8(6):e67445. doi: 10.1371/journal.pone.0067445 (PMC3691210; doi:10.1371/journal.pone.0067445)
Supplement: Table S10 — Nucleotide sequences of PCR primers used for cloning and sequencing of ospC alleles. (DOC) [file pone.0067445.s014.doc]

| **Primer name** | **Direction** | **Primer sequence (5’ to 3’)** | **Target OspC Type** | **Purpose** |
| --- | --- | --- | --- | --- |
| *ospC* Full Length | Fwd | ATGAAAAAGAATACATTAAGTGC | All | Cloning |
|  | Rev | ATTAATCTTATAATATTGATTTTAATTAAGG | All | Cloning |
| pXT7-ospC | Fwd | TATCGACGACGACGACAAGCATATGCTCGAGatggctaataattcaggaa*^a^* | All | Cloning |
|  | Rev1 | TTCCTTTCGGGCTTTGTTAGCAGCCGGATCttaaggtttttttggacttt | A, B, D, E, F, F3, H3, H, I, U, I3 | Cloning |
|  | Rev2 | TTCCTTTCGGGCTTTGTTAGCAGCCGGATCttaaggtttttttggagttt | A3, B3, C3, D3, E3, G, L, N, T | Cloning |
|  | Rev3 | TTCCTTTCGGGCTTTGTTAGCAGCCGGATCttaagggttttttggacttt | C, J, K | Cloning |
|  | Rev4 | TTCCTTTCGGGCTTTGTTAGCAGCCGGATCttaaggtttttttggatttt | M, O | Cloning |
| M13 | Fwd | GTAAAACGACGGCCA | Vector-specific | Sequencing |
|  | Rev | CAGGAAACAGCTATGAC | Vector-specific | Sequencing |
| pXT7-Sequencing | Fwd | TAATACGACTCACTATAGGGGAATTGT | Vector-specific | Sequencing |
|  | Rev | CCCCAAGGGGTTATGCTAGT | Vector-specific | Sequencing |

*^a^* For pXT7-*ospC* primers, nucleotides in lower case correspond to gene-specific sequence and upper case font nucleotides correspond to vector-specific sequences.
