# Supplementary material for: Inferring Epitopes of a Polymorphic Antigen Amidst Broadly Cross-Reactive Antibodies Using Protein Microarrays: A Study of OspC Proteins of Borrelia burgdorferi
Source: PLoS One. 2013 Jun 24;8(6):e67445. doi: 10.1371/journal.pone.0067445 (PMC3691210; doi:10.1371/journal.pone.0067445)
Supplement: Text S1 — Detailed information on Materials and Methods. (DOC) [file pone.0067445.s015.doc]

**Supplemental Text S1**

***Amplification and cloning of* ospC *alleles*** Table S9 provides the sources, geographic origins, accession numbers, and citations for the *ospC* alleles that were used for this study. Table S10 lists the name and nucleotide sequence of primers utilized to amplify genes for this study. Primers used for amplifying full-length *ospC* alleles were *ospC* Full Length Forward and Reverse, which sequences corresponded to positions 16903 through 16925 (Forward) and 17529 through 17558 (Reverse) of cp26 of the B31 strain. For amplification of subcloning targets for protein expression primers pXT7-*ospC* Forward and Reverse 1 through 4 were used. These primers contained a 30-mer sequence complimentary to the pXT7 vector and a 20-mer sequence complimentary to the *ospC* ORF. Primer pXT7-*ospC* Forward was the same for all reactions and corresponded to nucleotides 58 through 70 of B31 strain *ospC* including an additional Methionine and Alanine at the beginning of the ORF. This primer eliminated the signal peptide conserved amongst all *ospC* alleles. Primers pXT7-*ospC* Reverse 1 through 4 corresponded to nucleotide positions 614 through 633, and were used in different reactions to accommodate 3’ end nucleotide polymorphisms amongst *ospC* alleles. Primers used for sequencing of cloned and subcloned *ospC* alleles were M13 and pXT7-Sequencing, respectively.

For the PCR reaction mix, Phusion DNA polymerase and HF buffer (Finnzymes, Woburn, MA) in a final concentration of 200 μM of each deoxynucleoside triphosphate (dNTP) and 0.5 μM of PCR primers were used. For the reaction, a 3-minute denaturation step at 98°C was followed by 35 cycles at 98°C for 30 s, 50°C for 30 s, 72°C for 90 s, and a final extension at 72°C for 7 minutes.

***Nucleotide* sequence analysis** PCR products and plasmids were sequenced on both strands by Genewiz (La Jolla, CA) using forward and reverse primers. Analysis of nucleotide sequence was performed using SeqMan (http://www.dnastar.com) for sequence alignments, CodonCode Aligner (http://www.codoncode.com) for visual check of chromatograms and peak quality, and MacClade 4.08 (http://macclade.org) for final editing and display of alignments.

**Cloning and subcloning of ospC alleles** The PCR amplicons of full-length *ospC* alleles were cloned into pCR 2.1-TOPO vector, according to manufacturer’s instructions (Invitrogen, Carlsbad, CA), and the sequences were verified using vector specific M13 primers. *ospC* alleles cloned into TOPO vector (hereafter referred as TOPO-FL-*ospC*) matching *ospC* sequences published on Genbank were selected for subcloning into a pXT7 expression vector containing an amino-terminal 10X-Histidine (His) fusion tag, using the *in vivo* recombination cloning method [35]. To generate targets for subcloning, a second PCR reaction was performed using purified plasmid clones of TOPO-FL-*ospC* as template and PCR primers pXT7-*ospC* Forward and Reverse 1, 2, 3 or 4. These *ospC* amplicons were gel-purified with PureLink Quick Gel Extraction kit (Invitrogen, Carlsbad, CA).

For *in vivo* recombination cloning, 2 μl of gel-purified *ospC* amplicons with vector-specific overhangs were added to 40 ηg of linearized pXT7 vector and 10 μL of RecA^+^ DH5α *E. coli* cells. The mixture was incubated on ice for 45 min, followed by 1 min at 42°C, and incubated with 200 μL of SOC broth (Invitrogen, Carlsbad, CA) for 60 min at 37°C in an incubator rotating at 200 rpm. This mixture was added to 2 mL of Miller-modified LB broth medium (BD, Franklin Lakes, NJ) supplemented with 50 μg/mL of kanamycin and incubated over night at 37°C shaking at 250 rpm. Twenty microliters of culture were plated on Miller-modified LB agar (Fisher Scientific, Fair Lawn, NJ) with 50 μg/mL of Kanamycin, for colony selection. Transformants were picked and presence of *ospC* gene in the expression vector was verified by PCR. Plasmid of pXT7-*ospC* clones was isolated with PureLink HQ Mini Plasmid Purification kit (Invitrogen, Carlsbad, CA), and nucleotide sequence was verified using vector specific pXT7-Sequencing primers. Plasmids were then transformed into *E. coli* BL21(DE3)*pLysS* (Promega, Madison, WI) for protein expression.

**OspC protein expression and purification** BL21(DE3)*pLysS* *E. coli* cells transformed with pXT7-*ospC* plasmids were cultured in 10 mL Terrific Broth (MP Biomedicals, Solon, OH) with 50 μg/mL of kanamycin, at 37°C, shaking at 250 rpm with aeration until reaching OD_600_ 0.4-0.6. Recombinant protein expression was induced with IPTG (RPI, Mt. Prospect, IL) at a final concentration of 1 mM and incubation for additional 4 hours. Cells were harvested by centrifugation at 13000 rpm for 10 min at 4°C, the broth discarded and pellets frozen at -20 °C. Upon thawing, bacterial pellets were re-suspended in 1 mL BugBuster Protein Extraction Reagent supplemented with Benzonase Nuclease and EDTA-free Protease Inhibitor Cocktail (EMD Chemicals, Gibbstown, NJ) and lysed by gentle rocking on a platform for 20 min at 22°C. Cell lysates were spun down at 13000 rpm for 5 min and the supernadant containing His-tagged OspC fusion protein was collected.

Cleared bacterial lysate was incubated for 5 min at 22°C with 30 μl of Ni-coupled magnetic beads (MagneHis kit, Promega, Madison, WI). The mixture was placed on a magnetic stand and the lysate removed by suction. Magnetic beads bound to recombinant OspC protein were washed 3 times with 5 mL of wash buffer (30 mM Imidazole, 100 mM HEPES, pH 7.5). Purified proteins were eluted from MagneHis beads in elution buffer (500 mM Imidazole, 100 mM HEPES, pH 7.5), and buffer-exchanged into protein storage buffer (300 mM NaCl, 10 mM Tris-HCl, 2 mM EDTA, 5 mM β-Mercaptoethanol, pH 8.0) using Amicon Ultra-15 10,000 NMWL tubes (Millipore, Billerica, MA). Sample purity was estimated by densitometry of coomassie-blue stained protein bands on sodium dodecyl sulfate-polyacrylamide (SDS-PAGE) gels shown in Figure S4, using ImageJ (http://rsbweb.nih.gov/ij/). Protein concentration was determined by BCA Protein Assay kit (Pierce, Rockford, IL). Purified OspC protein samples were aliquoted and stored at -80°C until use.

**OspC protein array printing** Purified recombinant-OspC proteins were printed on nitrocellulose-coated glass FAST slides (Whatman, Piscataway, NJ) using an Omnigrid 100 apparatus (Digilab, Holliston, MA), in duplicate spots and 2 different amounts of approximately 10 pg and 30 pg of protein per spot. Protein storage buffer alone was printed on the array to serve as a background signal control.

**OspC protein microarray probing** Serum samples were diluted 1:200 (LD sera) or 1:100 (*P. leucopus*) in Protein Array Blocking (PAB) buffer (Whatman Inc, Sanford, ME) supplemented with 10% (vol/vol) DH5α *E. coli* lysate (MCLAB, San Francisco, CA). This suspension was incubated for 30 min at 22°C, with gentle constant shaking. Arrays were re-hydrated with PAB buffer for 30 min at 22°C. Primary antibody was placed on re-hydrated array pads and incubated for 12 h at 4°C on a rocker platform (Bellco, Vineland, NJ). Pads were rinsed 6 times, followed by three 5-minute washes in Tween-TBS. Cy3-conjugated secondary antibody, goat anti-human IgG heavy and light chain or goat anti-*Peromyscus leucopus* IgG heavy and light chain (KPL, Gaithersburg, MD), were diluted 1:200 in PAB buffer, and incubated with pads in the dark for 1 h at 22°C on a rocking platform. Arrays were washed in Tween-TBS as described above and this process repeated with TBS alone. Slides were dismounted from their frames, rinsed in double-distilled H_2_O and centrifuged 20 min at 500 x *g* to dry. Probed array slides were scanned in a Perkin Elmer ScanArray Express HT at a wavelength of 670 nm, at 95% laser power and 55% PMT. The output RGB TIFF files generated by the scanner were quantitated using ProScanArray Express software (Perkin Elmer, Waltham, MA) with spot-specific background correction [35].

**Abbreviations**

The notation and abbreviations defined here are used throughout the article:

*MSA*: multiple sequence alignment of OspC types containing both conserved and polymorphic positions

*MSA_poly_*: multiple sequence alignment of OspC types containing only polymorphic positions

*M_S-Global_*: matrix of pairwise global amino acid sequence identities between OspC types

*M_S-Local_*: matrix of pairwise local amino acid sequence identities between OspC types

*M_S-Local3D_*: matrix of pairwise amino acid sequence identities calculated using subsets of residues in close proximity in 3D space to a given central residue. Only polymorphic positions in *MSA_poly_* are used

*M_S-Nterm_*: matrix of pairwise local amino acid sequence identities between OspC types for an N-terminal sequence region

*M_S-Cterm_*: matrix of pairwise local amino acid sequence identities between OspC types for a C-terminal sequence region

*D_x_*: row of raw pixel intensity values for patient sera for OspC type *x*

*D_x-log_*: log_10_ values for patient sera for OspC type *x*

*D_x-rank_*: ranks for patient sera for OspC type *x*, i.e. the lowest raw reactivity value is replaced with 1 and the highest is replaced with 55

*D_x-binary_*: binary indicator values for patient sera for OspC type *x*. Values above the global median were set to 1 and values below were set to 0

*M_D_*: matrix of pairwise correlations of antibody binding intensity values between 2 OspC types calculated using patient sera, i.e. *M_D_*[OspC *x*][OspC *y*] = *r*(*D_x_ ,D_y_*). Also referred as antibody cross-reactivity matrix

*M_D-log_* : antibody cross-reactivity matrix calculated using log_10_–transformed values of antibody binding intensity

*M_D-rank_* : antibody cross-reactivity matrix calculated using rank values of antibody binding intensity

*M_D-binary_* : antibody cross-reactivity matrix calculated using binary values of antibody binding intensity
